# Antitumor effect and mechanism of an ellagic acid derivative on the HepG2 human hepatocellular carcinoma cell line

**DOI:** 10.3892/ol.2013.1740

**Published:** 2013-12-06

**Authors:** HUI ZHANG, ZENG-JUN GUO, WEN-MING XU, XIAO-JUAN YOU, LING HAN, YAN-XIA HAN, LIU-JIANG DAI

**Affiliations:** Faculty of Pharmacy, Medical College of Xi’an Jiaotong University, Xi’an, Shaanxi 710061, P.R. China

**Keywords:** antitumor activity, ellagic acid derivative, *Euphorbia hylonoma* Hand.-Mazz., human hepatoma HepG2

## Abstract

In the present study, to identify the effective components of Chinese traditional herbs, *Euphorbia hylonoma* Hand.-Mazz. (Euphorbiaceae), a folk herb that has been used among the Qinling mountain area for hundreds of years, was investigated. 3,3′-Di-O-methyl ellagic acid-4′-O-β-d-xylopyranoside (JNE2), an ellagic acid derivative, was isolated from the acetone extract of the herb and its antitumor activity against human hepatoma HepG2 cells was detected *in vitro*. The results showed that JNE2 inhibited the proliferation of HepG2 cells in a dose- and time-dependent manner and blocked the cell cycle at the G1/S phase. A high dosage of JNE2 induced apoptosis of the tumor cells, but no significant differences were identified between the treatment groups. The invasiveness of HepG2 cells was also inhibited by JNE2. The mechanism of the antitumor effect of JNE2 at the molecular level was presumed to be due to the upregulation of the protein expression of Bax and caspase-3, and the downregulation of the protein expression of Bcl-2 and CCND1. The results suggested that JNE2 is a potential antitumor agent that merits further investigation.

## Introduction

*Euphorbia hylonoma* Hand.-Mazz. (Euphorbiaceae), commonly known as Jiu Niuzao, has long been used as a folk medicine in China. It grows wildly in the Northwestern region of China and is used in antineoplastic intervention, as well as in the treatment of hepatocirrhosis, edema and incontinence (1). The bioactive and chemical constituents from the *Euphorbia* genus have been widely studied (2–7). It has been previously reported that several *Euphorbia* species are used in the treatment of skin diseases, gonorrhea, migraines (8) and cancerous conditions (9–11). Previous studies have reported the isolation of the chemical constituents from this plant, including tannins, ferulic acid esters and sesquiterpenoids (12,13). There have also been studies on the pharmacognosy of this plant (14,15).

However, studies concerning the antitumor components from this plant and their mechanisms of action are rare. In the current study, 3,3′-di-O-methyl ellagic acid-4′-O-β-d-xylopyranoside(JNE2), an ellagic acid derivative, was isolated from Euphorbiaceae during the anticancer screening study of traditional Chinese medicine (TCM). After analyzing its chemical features and comparing the spectral data with those of previous studies (16,17), this compound was identified as JNE2. The present study investigated the cytotoxic activity and the antitumor mechanisms of JNE2. Cell cycle, apoptosis and western blot analyses and cell invasion assay were employed, and the HepG2 human hepatoma cell line was adopted as the cell model.

## Materials and methods

### General materials

Nuclear magnetic resonance (NMR) spectra were recorded on a Bruker Avance III 500 NMR spectrometer (Bruker Corporation, Billerica, MA, USA) with tetramethylsilane as an internal standard. High-resolution electrospray ionization mass spectrometry was conducted using a Micromass Autospec-Ultima TOF mass spectrophotometer (Micromass UK Ltd., Altrincham, UK). The melting point was acquired using micro melting point apparatus (Beijing Tech Instrument Co., Ltd., Beijing, China). The materials used for column chromatography (CC) were silica gel (SiO_2_; 200–300 mesh; Qingdao Marine Chemical Factory, Qingdao, China) and Sephadex LH-20 (18–111 μm; GE Healthcare, Amersham, UK). Thin-layer chromatography (TLC) was conducted using glass precoated with silica gel (GF254; 10–40 mm; Qingdao Marine Chemical Factory).

### Plant material

The roots of *Euphorbia hylonoma* were collected from Qinling Mountain, Shaanxi, China, in September 2010 and were identified by Professor Juxian Lu, Faculty of Pharmacy, Medical College of Xi’an Jiaotong University (Xi’an, China). The voucher specimen was retained at the Department of Pharmacy, Medical School of Xi’an Jiaotong University for future reference.

### Cell culture

The HepG2 human hepatoma cell line was obtained from the Shanghai Institute of Biochemistry and Cell Biology, Chinese Academy of Sciences (Shanghai, China). HepG2 cells (5.0×10^4^ cells/ml) were cultured in RPMI-1640 supplemented with 10% fetal bovine serum (FBS), containing 2.0 mmol/l glutamine and 1% penicillin-streptomycin in 5% CO_2_ at 37°C, and were allowed to adhere for 24 h. The experiments were divided into the following five groups in the cell proliferation assay (MTT assay): Negative control (dimethyl sulfoxide; DMSO); positive control (15 μmol/l oxaliplatin); low-dosage (22.5 μmol/l JNE2); middle-dosage (45 μmol/l JNE2); and high-dosage (90 μmol/l JNE2). However, the low-dosage group was cut-off in other assays due to its low efficiency in the MTT assay.

### Extraction and isolation

**Figure 1:**
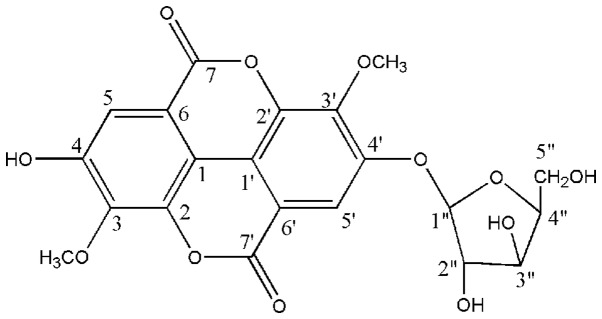
Chemical structure of 3,3′-di-O-methyl ellagic acid-4′-O-β-d-xylopyranoside.

The dried and powdered roots (1 kg) of *Euphorbia hylonoma* were extracted with acetone three times (24 h per extraction) at room temperature to obtain 212-g extracts. A portion of the acetone extracts (20 g) was chromatographed on a silica gel (500-g) column. The column was eluted with a gradient of petroleum ether-ethyl acetate (100:1 to 1:100) and methanol. The volume of each elution was 250 ml and underwent TLC inspection. The fractions with the same TLC spectrum behavior were combined to obtain seven fractions, A–G. Subsequently, fraction D (4.3 g) was further isolated on a silica gel column and eluted with petroleum-ether acetate (7:3). Further CC of subfraction B (1.2 g) from fraction D was performed on a Sephadex LH-20 column that was eluted with methanol. Compound JNE2 (0.6 g) was obtained from subfraction B-6. The identification of this compound was performed through the analysis of the spectroscopic features and comparing the spectral data with those of previous studies (16,17). This compound was identified as JNE2 (Fig. 1) as follows as follows: White powder, m.p. 241–243°C; ^1^H NMR (500 MHz, DMSO-d_6_) δ ppm: 7.48 (1H, d, *J*=5.3, H-5), 7.73 (1H, d, *J*=3.2, H-5), 4.05 (1H, s, 3-OCH_3_), 4.08 (1H, s, 3′-OCH_3_), 10.8 (1H, s, 4-OH); ^13^C NMR (125 MHz, DMSO-d_6_) δ ppm: 111.07 (C-1), 140.94(C-2), 140.19(C-3), 151.26 (C-4), 111.65 (C-5), 111.82 (C-6), 158.37 (C-7), 114.19 (C-1′), 141.58 (C-2′), 141.98 (C-3′), 152.84 (C-4′), 112.02(C-5′), 112.72(C-6′), 152.40 (C-7′), 101.94(C-1″), 73.09(C-2″), 76.17(C-3″), 69.31(C-4″), 65.84 (C-5″), 61.48(3-OCH_3_), 61.02 (3′-OCH_3_).

### 3-(4,5-Dimethylthiazol-2-yl)-2,5-diphenyltetrazolium bro mide (MTT) assay

HepG2 cells (2×10^4^ cells/well) were seeded in 96-well plates. Following overnight incubation, test substances were added and the incubation was continued at 37°C in an atmosphere containing 5% CO_2_ for 3 days. Subsequently, 20 μl MTT (Sigma-Aldrich, St. Louis, MO, USA) solution (5 g/l) was added into each well and incubated for an additional 4 h. Supernatants were removed and formazan crystals were dissolved in 200 μl DMSO. The optical density was measured at 490 nm using a POLARstar Optima (BMG Labtech GmbH, Ortenberg, Germany).

### Cell cycle and apoptosis analysis

Cell seeding and treatment were the same as MTT assay. After three days of treatment, cells were harvested by trypsinization and 1×10^6^ cells were counted and used for the analysis. Cells were fixed in ice-cold ethanol overnight at 4°C following washing with PBS. The cells were then washed in PBS again and incubated in 1 ml staining solution (20 μg/ml propidium iodide and 10 U/ml RNase A) for 30 min at room temperature. The cells were examined by fluorescence-activated cell sorting (FACS) using a flow cytometer (FACSort; Becton-Dickinson, Franklin Lakes, NJ, USA), and the cell cycle populations were determined using ModFit software (Verity Software House, Turramurra, Australia).

For the analysis of apoptotic cell populations, cells were trypsinized and washed in PBS. Staining with Alexa-Fluor 647 Annexin V (Invitrogen Life Technologies, Carlsbad, CA, USA) and propidium iodide was performed in 20 mmol/l HEPES buffer (pH 7.4), containing 150 mM NaCl and 2.5 mmol/l CaCl_2,_ for 15 min at room temperature. The cells were examined by FACS using a flow cytometer (FACSort; Becton-Dickinson), and the apoptotic populations were determined using ModFit software (Verity Software House).

### Cell invasion assay

Cell invasion was evaluated using the Chemicon QCM™ 24-well collagen-based cell invasion assay (Millipore, Billerica, MA, USA) according to the manufacturer’s instructions. In brief, 0.3 ml serum-free medium was added to the interior of each insert to rehydrate the collagen layer for 30 min at room temperature. The medium was then replaced with 0.3 ml prepared serum-free cell suspension containing 3.0×10^5^ cells and the corresponding test substances. Medium (500 μl) containing 10% FBS was added to the lower chamber and the cells were incubated for 24 h at 37°C. Following this, all non-invaded cells were removed from the interior of the insert and the invaded cells were stained with crystal violet. The stained cells were analyzed on an Olympus fluorescence microscope (BX43; Olympus Corporation, Tokyo, Japan).

### Western blot analysis

Cell seeding and treatment were the same as MTT assay. After three days of treatment, cells were harvested and washed in PBS. Cell protein lysates were separated in 10% SDS-polyacrylamide gels and electrophoretically transferred to polyvinylidene difluoride membranes (Roche Diagnostics, Mannheim, Germany). The lysates were then detected using rabbit polyclonal antibodies that were specific for Bcl-2, BAX, caspase-3 and CCND1 (Santa Cruz Biotechnology, Inc., Santa Cruz, CA, USA) and a commercial ECL kit (Pierce Biotechnology, Inc., Rockford, IL, USA). Protein loading was estimated by human anti-β-actin monoclonal antibody (Santa Cruz Biotechnology, Inc.).

### Statistical analysis

Statistical analysis was performed by one-way ANOVA test followed by Fisher’s protected least significant difference post hoc test for multiple comparisons using the StatView program (Abacus Concepts, Berkeley, CA, USA). P<0.05 was considered to indicate a statistically significant difference.

## Results

### Cell growth inhibition

**Figure 2:**
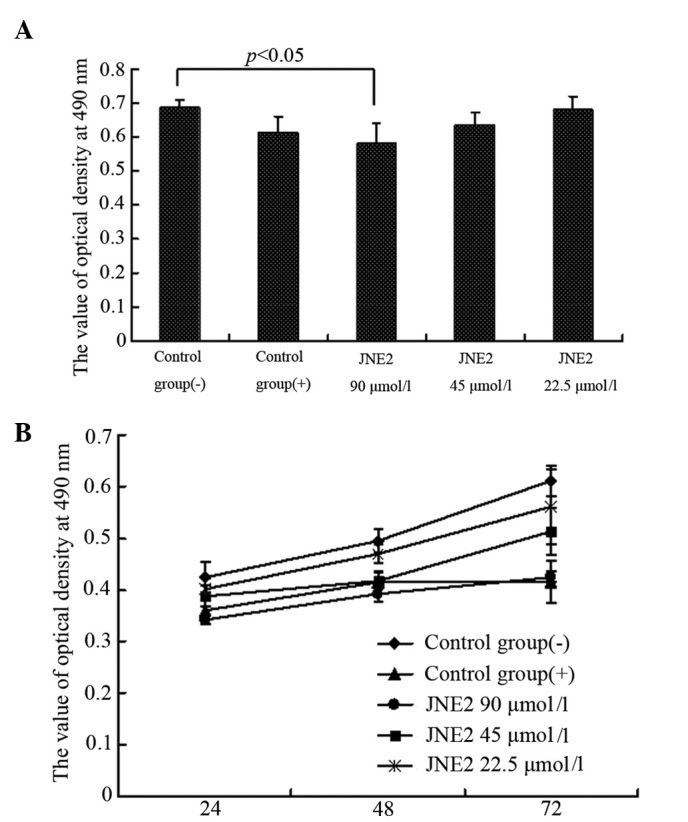
Growth inhibitory effect of JNE2 on HepG2 cells. (A) Dose-dependent effect of JNE2 on HepG2 cells after 24 h and (B) time-dependent effect of JNE2 on HepG2 cells after 3 days when the cell viability was determined by 3-(4,5-dimethylthiazol-2-yl)-2,5-diphenyltetrazolium bromide assay. JNE2, 3,3′-di-O-methyl ellagic acid-4′-O-β-d-xylopyranoside.

**Figure 3:**
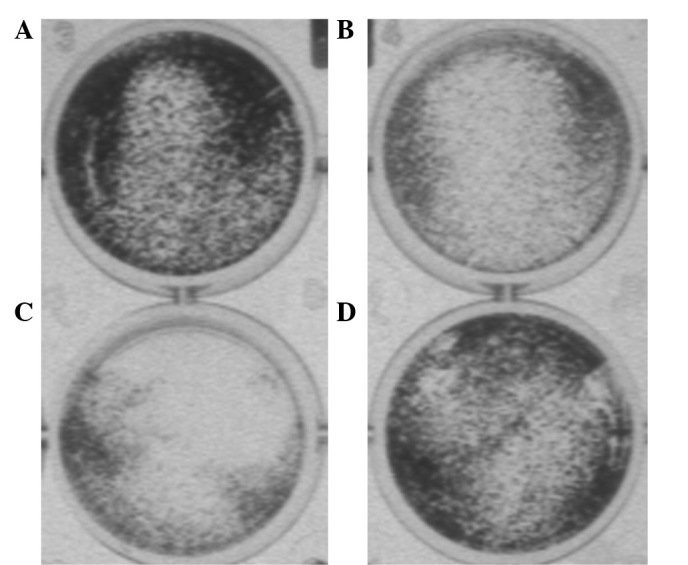
Colony formation of HepG2 cells following treatment with (A) Dimethyl sulfoxide, (B) oxaliplatin and (C) 90 and (D) 45 μmol/l of 3,3′-di-O-methyl ellagic acid-4′-O-β-d-xylopyranoside for 3 days.

To evaluate the antitumor role of JNE2 on human hepatoma cells, MTT and colony formation assays were employed to detect the growth of HepG2 cells at various time points following treatment with JNE2 at various concentrations. The results showed that JNE2 exhibited a growth inhibitory effect on HepG2 cells in a dose- and time-dependent manner. Following 24 h of treatment, the OD value of the high-dosage group (JNE2, 90 μmol/l) was significantly lower than that of the negative control group (P<0.05) and also lower than that of the positive control group, but without significant difference (Fig. 2A). The time-effect curve also demonstrated the antiproliferation ability of JNE2 (Fig. 2B). Furthermore, the colony formation results shown in Fig. 3 confirmed that a high dosage of JNE2 may inhibit the growth of HepG2 cells. These results suggested that JNE2 exhibits an inhibitory effect on the proliferation of hepatoma cells.

### JNE2-induces G0/G1 cell cycle arrest

**Figure 4:**
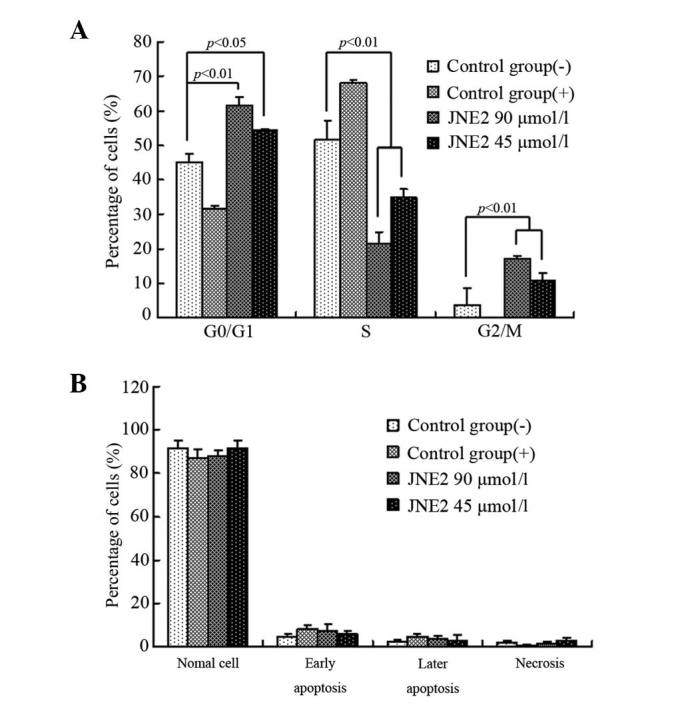
Effect of JNE2 on the cell cycle and apoptosis of HepG2 cells. (A) JNE2-induced G0/G1 cell cycle arrest in HepG2 cells. Effects of JNE2 on the cell cycle distribution following 24 h of treatment with various concentrations are presented. (B) JNE2-induced apoptosis of human hepatoma HepG2 cells following 24 h of treatment. The percentages of normal, early and late apoptotic and necrotic cells are presented. JNE2, 3,3′-di-O-methyl ellagic acid-4′-O-β-d-xylopyranoside.

To explore the mechanism underlying JNE2-suppressed cell proliferation, the impact of JNE2 on cell cycle progression was investigated by FACS and the results are presented in Fig. 4A. Following treatment with high and middle dosages of JNE2, the cell cycle shifted from a high S phase population to a high G1 phase population, together with an accumulation of a G2/M phase population. Whereas, little effect on the cell cycle was observed in the control groups. These results indicated that JNE2 blocks the G1/S transition.

### JNE2-induces HepG2 cell apoptosis

To examine the effect of JNE2 on apoptosis, hepatoma cells were treated with various concentrations of JNE2. Compared with the cells that were treated with the negative control, the cells that were treated with a high dosage of JNE2 exhibited a similar apoptotic rate to those treated with oxaliplatin, whereas the cells that were treated with 45 μmol/l JNE2 exhibited slightly lower apoptotic rates. In the positive and JNE2-treated groups, the number of cells at the early and late stages of apoptosis marginally increased. However, no significant differences were identified (Fig. 4B) between the groups. These results showed that JNE2 induces apoptosis in human hepatoma cells in a dose-dependent manner.

### JNE2 inhibits cancer invasion in vitro

**Figure 5:**
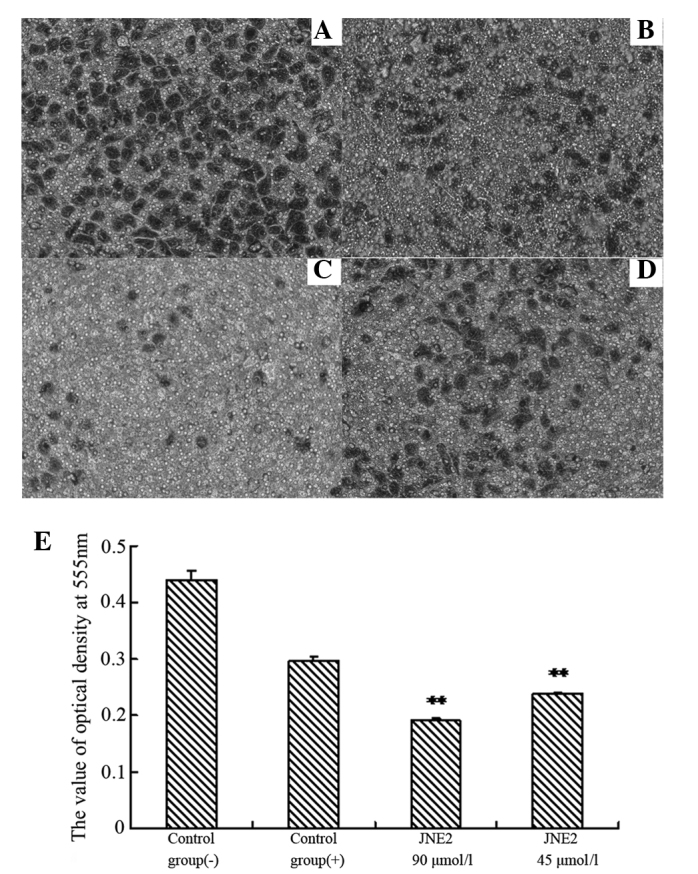
Invasion inhibition effect of JNE2 on HepG2 cells. (A) Control group (−), (B) control group (+) and (C) 90 and (D) 45 μmol/l JNE2-treated groups. (E) OD values of various groups following 24 h treatment with JNE2 or control. ^**^P<0.01, vs. negative control.. JNE2, 3,3′-di-O-methyl ellagic acid-4′-O-β-d-xylopyranoside.

Cancer invasion is the process by which cells break away from the primary tumor and migrate through the surrounding tissue. This enables the cancer cells to move into blood vessels and travel through the body, possibly establishing a secondary tumor at an additional site (18). To determine whether JNE2 inhibits invasion, HepG2 cells were treated with various concentrations of JNE2 and the control groups were treated separately. Evidently, the invasion was inhibited by a high concentration of JNE2, whereas, migration was not altered in the cells that were treated with the negative control (Fig. 5). These results markedly suggest that JNE2 inhibits the invasion of HepG2 cells.

### Western blot analysis

**Figure 6:**
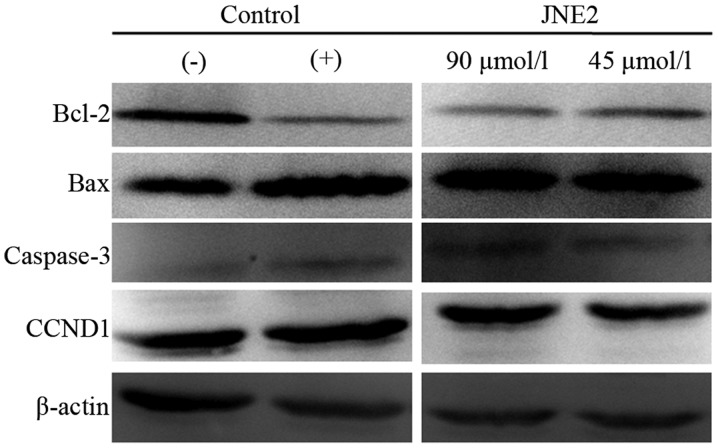
Regulatory effects of JNE2 on apoptosis-related proteins in HepG2 cells. JNE2, 3,3′-di-O-methyl ellagic acid-4′-O-β-d-xylopyranoside.

To clarify the apoptotic mechanism of JNE2 on HepG2 cells, western blot analysis was performed to examine the protein expression levels of Bcl-2, Bax, caspase-3 and CCND1 (cyclin D1). The cells that were treated with JNE2 exhibited upregulated levels of Bax and caspase-3 expression when compared with the negative control cells. This result may explain why JNE2 inhibited the growth of HepG2 cells, and this function may be associated with mitochondrial pathway-induced apoptosis. Conversely, the protein levels of Bcl-2 and CCND1 were decreased with JNE2 treatment (Fig. 6). The apoptotic effect of JNE2 was confirmed by the upregulation of Bax and downregulation of Bcl-2. Based on the G0/G1 arresting capability, the downregulation of CCND1 may provide an explanation. These results suggested that JNE2 may exhibit its apoptotic effect through the upregulation of Bax and caspase-3, and the downregulation of Bcl-2 and CCND1.

## Discussion

Ellagic acid, a type of polyphenol compound that widely exists in herbs and numerous types of fruits and nuts, has recently gained increasing attention, although it was once considered useless in TCM. It has been well established that ellagic acid exhibits anticancer (19), antimutagen (20–22) and antimicrobial functions (23), as well as others. Numerous studies concerning the antimutagen and anticancer effects of ellagic acid were performed in the 1970s (24–27). In previous *in vitro* and *in vivo* experiments, ellagic acid has shown significant abilities in inhibiting the growth of a number of types of tumor, such as skin, esophagus and lung, as well as other tumors that are caused by carcinogens (28–32). Its anticancer activities are partially based on the quenching of reactive oxygen species, thereby protecting critical cellular targets (i.e. DNA, proteins and lipids) from oxidative insult (33–35). Ellagic acid may also interfere with intracellular signaling pathways, such as those that regulate proliferation, induce apoptosis and respond to oxidative stress (36–39). In the present study, JNE2, an ellagic acid derivative that was isolated from a traditional Chinese herb, *Euphorbia hylonoma* Hand.-Mazz. (Euphorbiaceae), showed significant antitumor effects on the HepG2 human hepatocellular carcinoma cell line. The antitumor mechanisms of JNE2 were also investigated.

The results of the current study showed that JNE2 may inhibit the proliferation of HepG2 cells. Specifically, JNE2 affected the cell cycle by arresting the cells in the G0/G1 phase, thus, inducing apoptosis. Cancer cell invasion was also inhibited by JNE2, particularly at a high concentration (90 μmol/l). Overall, these results demonstrated the outstanding antitumor ability of JNE2. The regulatory effects of JNE2 upon apoptosis-related protein targets, including Bcl-2, Bax, caspase-3 and CCND1, were measured to determine its antitumor mechanism at the molecular level.

Bcl-2 family proteins that comprise proapoptotic proteins (such as Bax, Bad and Bid) and antiapoptotic proteins (such as Bcl-2 and Bcl-xL) tightly regulate the mitochondrial apoptosis pathway. A number of anticancer drugs trigger mitochondria-mediated apoptosis in cancer cells through the downregulation of Bcl-2/Bcl-xL or the upregulation of Bax/Bad/Bid. Bcl-2 is an antiapoptosis gene (40–44) and is closely associated with cellular apoptosis (45,46), as well as with the mitochondrion (47,48). Bax induces cellular apoptosis and the ratio of Bcl-2/Bax is the determining factor of antiapoptosis for cells (49). The results of the present study showed that JNE2 treatment significantly upregulated the expression of Bax protein and downregulated that of Bcl-2. This suggested that JNE2 acts on the Bcl-2/Bax genes to exert its apoptotic effect.

Caspases, a family of cysteine acid proteases, act as important mediators of apoptosis. They are induced by various stimuli and contribute to the overall apoptotic phenotype by cleaving various cellular substrates (50–52). Caspase-3, a key regulatory protease upon which a number of signaling pathways merge for the execution of apoptosis, is involved in apoptosis induced by Bcl-2/Bax, p38 and JAK-STAT (53,54). In the current study, the protein expression of caspase-3 was detected in HepG2 cells following treatment with JNE2 and the upregulated effect was identified. These results suggested that JNE2 induced the apoptosis of human hepatoma HepG2 cells via the mitochondrial pathway.

CCND1 is a cell cycle control protein that mainly affects G1 progression and G1/S transition. It forms complexes with CDK4 and CDK6, and additionally with RB1. The phosphorylation of RB1 by CCND1/CDK4 prevents cell cycle arrest at the G1/S start point. The overexpression of CCND1 causes oncogenesis due to its stimulation of the expression of Bcl-1, which accelerates cell transition through the G1 phase. Therefore, JNE2 shows the ability to block the G1/S phase transition by downregulating the expression of CCND1.

However, among the downregulated and upregulated expression ratios of Bcl-2/Bax, caspase-3 and CCND1, the predominant mechanism by which JNE2 induces mitochondria-mediated apoptosis in HepG2 cells is uncertain and other regulatory mechanisms, including at the receptor level, require further investigation.

## References

[1] Jiangsu New Medical College (1993). Dictionary Traditional Drugs.

[2] Whelan LC, Ryan MF (2003). Ethanolic extracts of *Euphorbia* and other ethnobotanical species as inhibitors of human tumour cell growth. Phytomedicine.

[3] Bruni R, Muzzoli M, Ballero M, Loi MC, Fantin G, Poli F, Sacchetti G (2004). Tocopherols, fatty acids and sterols in seeds of four Sardinian wild *Euphorbia* species. Fitoterapia.

[4] Hore SK, Ahuja V, Mehta G, Kumar P, Pandey SK, Ahmad AH (2006). Effect of aqueous *Euphorbia hirta* leaf extract on gastrointestinal motility. Fitoterapia.

[5] Shi HM, Williams ID, Sung HH, Zhu HX, Ip NY, Min ZD (2005). Cytotoxic diterpenoids from the roots of *Euphorbia ebracteolata*. Planta Med.

[6] Ruan HL, Zhang Y, Zhang YH (2006). Studies on constituents from roots of *Euphorbia hylonoma*. Zhongguo Zhong Yao Za Zhi.

[7] Cao D, SU YL, Yang JS (1996). Triterpene constituents from *Euphorbia nemtocypha Hand*-Mazz. Yao Xue Xue Bao.

[8] Chaabi M, Michel VF, Frossard N, Randriantsoac A, Andriantsitohainad R, Lobstein A (2007). Anti-proliferative effect of *Euphorbia stenoclada* in human airway smooth muscle cells in culture. J Ethnopharmacol.

[9] Valente C, Pedro M, Duarte A, Nascimento MS, Abreu PM, Ferreira MJ (2004). Bioactive diterpenoids, a new jatrophane and two ent-Abietanes, and other constituents from *Euphorbia pubescens*. J Nat Prod.

[10] Zhang WF, Cui Z, Zhu Q (1998). Cytotoxicity and antiviral activity of the compounds from *Euphorbia kansui*. Planta Med.

[11] Yasukawa K, Akihisa T, Yoshida ZY, Takido M (2000). Inhibitory effect of euphol, a triterpene alcohol from the roots of *Euphorbia kansui*, on tumor promotion by 12-O-Tetradecanoylphorbol-13-acetate in two-stage carcinogenesis in mouse skin. J Pharm Pharmacol.

[12] Guo ZJ, Zhu R, Lu JX, Li YL (1995). Chemical constituents of *Euphorbia hylonoma* Hand. -Mazz. Zhongguo Zhongyao Zazhi.

[13] Ruan HL, Zhou XF, Zhang YH, Pi HF, Wu JZ, Sun HD (2007). Ferulic acid esters from *Euphorbia hylonoma*. Fitoterapia.

[14] Guo ZJ, Lu Juxian, Li YL (1996). Studies on pharmacognosy of *Euphorbia hylonoma* Hand-Nazz. J Xi’an Med Univ.

[15] Guo ZJ, Lu X, Li YL, Zhu R (1996). The studies on resource of *Euphorbia* genus in Shaanxi province. Northwest Pharm J.

[16] Liu RH, Chen LL, Kong LY (2002). Ellagic Acid Derivatives from the Stem Bark of *Sapius sebiferum*. Zhong Guo Yao Ke Da Xue Xue Bao Bian Ji Bu.

[17] Hong YX, Wei GY (2004). Studies on the chemical constituents from leaves of *Diplopanax stachyathus*. Zhong Cao Yao Bian Ji Bu.

[18] Duffy MJ, McGowan PM, Gallagher WM (2008). Cancer invasion and metastasis: changing views. J Pathol.

[19] Maas JL, Galletta GJ (1991). Ellagic acid, an anticarcinogen in fruits, especially in strawberries: a review. Hortscience.

[20] Thresiamma KC, George J, Kuttan R (1998). Protective effect of curcumin, ellagic acid and bixin on radiation induced genotoxicity. J Exp Clin Cancer Res.

[21] Mandal S, Stoner GD (1990). Inhibition of N-nitrosobenzylmethylamine-induced esophageal tumorigenesis in rats by ellagic acid. Carcinogenesis.

[22] Sohn EH, Koo HJ, Hang DT (2013). Protective effects of ellagic acid on ethanol-induced toxicity in hepatic HepG2 cells. Mol Cell Toxicol.

[23] Lu HF, Tsou MF, Lin JG, Ho CC, Liu JY, Chuang JY, Chung JG (2005). Ellagic acid inhibits growth and arylamine N-acetyltransferase activity and gene expression in *Staphylococcus aureus*. In Vivo.

[24] Sayer JM, Yaji H, Wood AW (1982). Extremely facile reaction between the ultimate carcinogen benzo[a]pyrene-7,8-diol 9,10-epoxide and ellagic acid. J Am Chem Soc.

[25] Take Y, Imouye Y, Nakamura S (1989). Comparative studies of the inhibitory properties of antibiotics on human immunodeficiency virus and avian myeloblastosis virus reverse transcriptases and cellular DNA polymerases. J Antibiot (Tokyo).

[26] Stoner GD, Morse MA (1997). Isothiocyanates and plant polyphenols as inhibitors of lung and esophageal cancer. Cancer Lett.

[27] Narayanan BA, Geoffroy O, Willingham MC, Re GG, Nixon DW (1999). P53/p21 (WAF1/CIP1) expression and its possible role in G1 arrest and apoptosis in ellagic acid treated cancer cells. Cancer Lett.

[28] Ahn D, Putt D, Kresty L, Stoner GD, Fromm D, Hollenberg PF (1996). The effects of dietary ellagic acid on rat hepatic and esophageal mucosal cytochromes P450 and phase II enzymes. Carcinogenesis.

[29] Falsaperla M, Morgia G, Tartarone A, Ardito R, Romano G (2005). Support ellagic acid therapy in patients with hormone refractory prostate cancer (HRPC) on standard chemotherapy using vinorelbine and estramustine phosphate. Eur Urol.

[30] Labrecque L, Lamy S, Chapus A (2005). Combined inhibition of PDGF and VEGF receptors by ellagic acid, a dietary-derived phenolic compound. Carcinogenesis.

[31] Arulmozhi V, Pandian K, Mirunalini S (2013). Ellagic acid encapsulated chitosan nanoparticles for drug delivery system in human oral cancer cell line (KB). Colloids Surf B Biointerfaces.

[32] Rommel A, Wrolstad RE (1993). Ellagic acid content of red rasp2 berry juice as influenced by cultivar, processing, and environ2 mental factors. J Agric Food Chem.

[33] Rafter JJ (2002). Scientific basis of biomarkers and benefits of functional foods for reduction of disease risk: cancer. Br J Nutr.

[34] Potter JD (1997). Cancer prevention: epidemiology and experiment. Cancer Lett.

[35] Roy M, Chakrabarty S, Sinha D, Bhattacharya RK, Siddiqi M (2003). Anticlastogenic, antigenotoxic and apoptotic activity of epigallocatechin gallate: a green tea polyphenol. Mutat Res.

[36] Kong AN, Owuor E, Yu R, Hebbar V, Chen C, Hu R, Mandlekar S (2001). Induction of xenobiotic enzymes by the MAP kinase pathway and the antioxidant or electrophile response element (ARE/EpRE). Drug Metab Rev.

[37] Park AM, Dong Z (2003). Signal transduction pathways: targets for green and black tea polyphenols. J Biochem Mol Biol.

[38] Loo G (2003). Redox-sensitive mechanisms of phytochemical mediated inhibition of cancer cell proliferation. J Nutr Biochem.

[39] Heber D, Lu QY (2002). Overview of mechanisms of action of lycopene. Exp Biol Med (Maywood).

[40] O’Neill JW, Hockenbery DM (2003). Bcl-2-related proteins as drug targets. Current Med Chem.

[41] Cory S, Adams JM (2005). Killing cancer cells by flipping the Bcl-2/Bax switch. Cancer Cell.

[42] Fathi NA, Hussein MR, Hassan HI, Mosad E, Galal H, Afifi NA (2006). Glomerular expression and elevated serum Bcl-2 and Fas proteins in lupus nephritis: preliminary findings. Clin Exp Immunol.

[43] Zhang R, Xue YY, Lu SD, Wang Y, Zhang LM, Huang YL, Signore AP, Chen J, Sun FY (2006). Bcl-2 enhances neurogenesis and inhibits apoptosis of newborn neurons in adult rat brain following a transient middle cerebral artery occlusion. Neurobiol Dis.

[44] Karlnoski R, Wilcock D, Dickey C, Ronan V, Gordon MN, Zhang W, Morgan D, Taglialatela G (2007). Up-regulation of Bcl-2 in APP transgenic mice is associated with neuroprotection. Neurobiol Dis.

[45] Bernas T, Asem EK, Robinson JP, Cook PR, Dobrucki JW (2005). Confocal fluorescence imaging of photosensitized DNA denaturation in cell nuclei. Photochem Photobiol.

[46] Yoshida A, Takemura H, Inoue H, Miyashita T, Ueda T (2006). Inhibition of glutathione synthesis overcomes Bcl-2-mediated topoisomerase inhibitor resistance and induces nonapoptotic cell death via mitochondrial-independent pathway. Cancer Res.

[47] Degli EM (2004). Mitochondria in apoptosis: past, present and future. Biochem Soc Trans.

[48] Dias N, Bailly C (2005). Drugs targeting mitochondrial functions to control tumor cell growth. Biochem Pharmacol.

[49] Sedlak TW, Oltvai ZN, Yang E, Wang K, Boise LH, Thompson CB, Korsmeyer SJ (1995). Multiple Bcl-2 family members demonstrate selective dimerizations with Bax. Proc Natl Acad Sci USA.

[50] Riedl SJ, Shi Y (2004). Molecular mechanisms of caspase regulation during apoptosis. Nat Rev Mol Cell Biol.

[51] Nunẽza G, Benedict MA, Hu Y, Inohara N (1998). Caspases: the proteases of the apoptotic pathway. Oncogene.

[52] Fan TJ, Han LH, Cong RS, Liang J (2005). Caspase family proteases and apoptosis. Acta Biochim Biophys Sin (Shanghai).

[53] Dassé E, Bridoux L, Baranek T, Lambert E, Salesse S, Sowa ML, Martiny L, Trentesaux C, Petitfrère E (2007). Tissue inhibitor of metalloproteinase-1 promotes hematopoietic differentiation via caspase-3 upstream the MEKK1/MEK6/p38alpha pathway. Leukemia.

[54] Lanvin O, Gouilleux F, Mullié C, Mazière C, Fuentes V, Bissac E, Dantin F, Mazière JC, Régnier A, Lassoued K, Gouilleux-Gruart V (2004). Interleukin-7 induces apoptosis of 697 pre-B cells expressing dominant-negative forms of STAT5: evidence for caspase-dependent and -independent mechanisms. Oncogene.

